# Modifying patterns of movement in people with low back pain -does it help? A systematic review

**DOI:** 10.1186/1471-2474-13-169

**Published:** 2012-09-07

**Authors:** Robert A Laird, Peter Kent, Jennifer L Keating

**Affiliations:** 1 Department of Physiotherapy, Monash University, PO Box 527, Frankston, Victoria, 3199, Australia; 2 Research Department, Spine Centre of Southern Denmark, Lillibaelt Hospital, Institute of Regional Health Services Research, University of Southern Denmark, Middelfart, Denmark; 3 Postal Address: 7 Kerry Rd, Warranwood, Melbourne, Victoria, 3134, Australia

**Keywords:** Low back pain, Movement disorders, Randomized controlled trial, Exercise therapy, Posture

## Abstract

**Background:**

Physiotherapy for people with low back pain frequently includes assessment and modification of lumbo-pelvic movement. Interventions commonly aim to restore normal movement and thereby reduce pain and improve activity limitation. The objective of this systematic review was to investigate: (i) the effect of movement-based interventions on movement patterns (muscle activation, lumbo-pelvic kinematics or postural patterns) of people with low back pain (LBP), and (ii) the relationship between changes in movement patterns and subsequent changes in pain and activity limitation.

**Methods:**

MEDLINE, Cochrane Central, EMBASE, AMI, CINAHL, Scopus, AMED, ISI Web of Science were searched from inception until January 2012. Randomised controlled trials or controlled clinical trials of people with LBP were eligible for inclusion. The intervention must have been designed to influence (i) muscle activity patterns, (ii) lumbo-pelvic kinematic patterns or (iii) postural patterns, and included measurement of such deficits before and after treatment, to allow determination of the success of the intervention on the lumbo-pelvic movement. Twelve trials (25% of retrieved studies) met the inclusion criteria. Two reviewers independently identified, assessed and extracted data. The PEDro scale was used to assess method quality. Intervention effects were described using standardised differences between group means and 95% confidence intervals.

**Results:**

The included trials showed inconsistent, mostly small to moderate intervention effects on targeted movement patterns. There was considerable heterogeneity in trial design, intervention type and outcome measures. A relationship between changes to movement patterns and improvements in pain or activity limitation was observed in one of six studies on muscle activation patterns, one of four studies that examined the flexion relaxation response pattern and in two of three studies that assessed lumbo-pelvic kinematics or postural characteristics.

**Conclusions:**

Movement-based interventions were infrequently effective for changing observable movement patterns. A relationship between changes in movement patterns and improvement in pain or activity limitation was also infrequently observed. No independent studies confirm any observed relationships. Challenges for future research include defining best methods for measuring (i) movement aberrations, (ii) improvements in movements, and (iii) the relationship between changes in how people move and associated changes in other health indicators such as activity limitation.

## Background

The causes of low back pain (LBP) appear to be complex and multifactorial, with both biological and psychosocial components associated with chronicity [1,2]. While numerous patho-anatomic structures have been associated with LBP, it is often difficult to establish a definitive anatomical cause or initiating factor for LBP in individual people [3,4]. Furthermore, although the pathogenesis of LBP has also been associated with genetic causes [5], such influences are not readily modifiable. In daily practice, many clinicians observe and treat physical impairments ranging from postural anomalies [6,7], localised intervertebral kinetic disturbance [8], motor control disturbance [9,10], muscle imbalance [11] and muscle atrophy [12].

People with persistent (chronic) or recurrent LBP have been variably reported to exhibit movement pattern aberrations such as increased trunk stiffness [9,13], poor proprioception [14], altered patterns of activation of abdominal muscles [10,15], extensor muscles [16-18], and postural dysfunction [19-21]. Different patterns of lumbo-pelvic kinematics during activities such as forward bending and sit-to-stand have been demonstrated in studies comparing people with and without LBP [22-25]. Methods for measuring lumbo-pelvic movement patterns can by categorised into three broad target groups: (i) muscle activity patterns, for example the contribution of deep versus superficial trunk muscles, (ii) patterns of hip to lumbar kinematics, for example the relative contributions of hip joint compared with lumbar spine movement to specific activities such as forward bending or walking, and (iii) postural patterns, for example slumped sitting compared with upright sitting posture.

Numerous interventions have targeted movement pattern aberrations associated with chronic LBP [10,26-29]. Some exercise interventions involve whole body movements such as aerobic exercise, Pilates, and yoga, while others target the activity of specific muscles. The effectiveness of exercise for LBP appears modest and not consistently associated with any particular form of exercise [30-32]. No consistent differences in LBP outcomes have been observed for highly individualised exercise programs that aim to alter lumbo-pelvic kinematics or postural patterns such as those based on the Alexander Technique [33,34], the Feldenkrais Method [33] or Pilates [35] compared with non-specific exercise. Similarly, reviews of interventions designed to alter patterns of specific muscle activity, variably described as motor control, trunk stabilisation or core stabilising exercise, have concluded little difference between outcomes achieved with motor control exercise compared with general exercise regimens [36-40]. As there is no standardisation in the reporting of exercise type, intensity, duration or frequency, one possibility is that some exercises are effective, but when trial outcomes are pooled, method heterogeneity in included studies precludes identification of trial-specific effectiveness.

Movement pattern aberrations associated with LBP, such as deviation from the normal activation patterns of Transversus Abdominus (TA) [10,41] have been reported. However the effect of interventions on these aberrant movement deficits has not been systematically evaluated. While most trials report effects on pain or activity limitation, few have measured changes in movement or postural patterns. This is reflected in five recent systematic reviews on the effectiveness of stabilisation (‘motor control’) exercises for LBP [36-40], which collectively synthesised 26 randomised controlled trials. More than half of the included trials in these reviews [36-40] used outcome measures of pain and activity limitation without measurement of any movement characteristic. Only three of 26 trials measured the effect of the intervention on a specific movement pattern aberration. As few trials measure movement pattern aberrations, this leaves three fundamental questions unanswered by existing reviews: (i) were movement pattern aberrations actually present in trial participants who received interventions designed to remedy these deficits? (ii) did the intervention achieve the intention of changing the movement pattern? and (iii) were improvements in other health parameters such as pain and activity limitation related to changes in movements classified as aberrant? To understand whether treatment can change movement pattern aberration, measurement of such deficits should occur before and after treatment, and the outcomes compared with those of a control group.

### Aims of this review

The first aim of this systematic review was to determine the effect of movement-based interventions on movement patterns defined as physical measures of muscle activation, lumbo-pelvic kinematics or postural patterns in adults with LBP. The second aim was to examine the relationship between changes in movement patterns and subsequent changes in pain and activity limitation.

## Methods

### Data sources

Eight electronic databases (MEDLINE, Cochrane CENTRAL, EMBASE, AMI, CINAHL, Scopus, AMED, ISI Web of Science) were searched from inception until January 2012 using a sensitive search strategy based on that recommended by the Cochrane Collaboration (included as an Additional File). The search yield was initially screened for eligibility by one reviewer (RL) on title and abstract to remove duplicates and clearly unrelated articles. A more detailed screening on title and abstract, and subsequently on retrieved full text articles, was performed independently by two reviewers (RL and PK). Disagreements were resolved by discussion. The protocol for this review has not previously been registered or published.

### Study selection: inclusion and exclusion criteria

Trials were included if they were randomised controlled trials or controlled clinical trials that only contained participants with lumbo-pelvic pain (+/− leg pain) in both the intervention and control groups. The intervention must have been specifically designed to influence any one of three observable patterns of movement: (i) muscle activity patterns, (ii) lumbo-pelvic kinematic patterns or (iii) postural patterns. To be as inclusive as possible, no restrictions were placed on the duration of complaint or pain location. Full inclusion details of each study are provided in Additional file 1: Appendix 1. Exclusion criteria were trials of animals, of drug interventions and trials that included people who were pregnant or had spinal malignancy, infection, fracture, cauda equina syndrome, metabolic or spinal inflammatory disorders.

### Types of outcome measures

For trials to be included, pre- and post-intervention data that quantified baseline measures and the effect on the target movement pattern relative to control measurements must have been reported. In the absence of these data, it could not be determined if the intervention was effective in changing the physical parameter it was designed to influence. These data were also required to investigate the relationship between change in movement patterns and change in health outcomes (pain and activity limitation). Acceptable methods for assessing movement patterns included any measures of specific muscle activation (eg timing of contraction, cross-sectional area, muscle thickness, electromyographic activity, ultrasound or other imaging measurement), lumbo-pelvic kinematics (eg a change in sequence, timing or coordination of movements such as lumbar versus hip contribution during lifting, sit-to-stand, forward bending) and any measures of sustained positions/postures of the lumbo-pelvic region (eg analysis of spinal kinematics within specified activities such as standing, sitting or sustained bending). Data must have been provided that described movement patterns (e.g. hip versus lumbar range, deep versus superficial muscle activity, particular sequences of timing, electrical activity or movement etc.).

Exclusion criteria at the level of outcome type were trials with outcomes that described only global range of movement or global measures of strength (eg trunk extension range or strength only), or trials that did not include data that enabled estimates of change in pain or activity limitation. This was because we considered that global range or strength were not surrogate measures of how the body coordinates movement patterns.

### Data extraction

From all included papers, two assessors independently extracted the following data: compliance with review inclusion criteria, type and duration of intervention for experimental and comparison groups, number and type of participants, the targeted movement characteristic (muscle activity pattern, lumbo-pelvic kinematic pattern or postural pattern), pre- and post-intervention outcome measurements and their method of measurement. Data extracted by these reviewers (RL and PK) were checked for concordance and where differences occurred, a third reviewer (JK) cross-checked data with consensus reached by discussion.

### Assessment of method quality

The PEDro scale was applied to assess potential sources of bias in included studies [42]. The PEDro scale has been reported as being adequately reliable [43] and valid [44]. Each clinical trial with a quality rating score on the PEDro website (http://www.pedro.org.au) has been independently assessed by two raters trained to assess method quality. Therefore where available, we used the quality scores from the PEDro website for included trials. There were two trials (reported in three papers) where scores were not available [45-47] and these were independently assessed (RL and PK) using the same PEDro scale and decision rules.

### Data synthesis and analysis

Study details (inclusion/exclusion criteria, intervention and comparison treatments and outcome measure details) were extracted and summarized (see Additional file 1: Appendix 2). Means and standard deviations (SDs) for intervention and control groups, for each comparison, at each reported outcome period and for all three categories of outcome variables (movement pattern, pain, activity limitation) were entered into Revman (v5) software [48]. This software was used to calculate standardised mean differences (SMD) between intervention and comparison groups. Negative values for SMDs indicated outcomes in favour of the experimental group.

## Results

### Search yield

**Figure 1:**
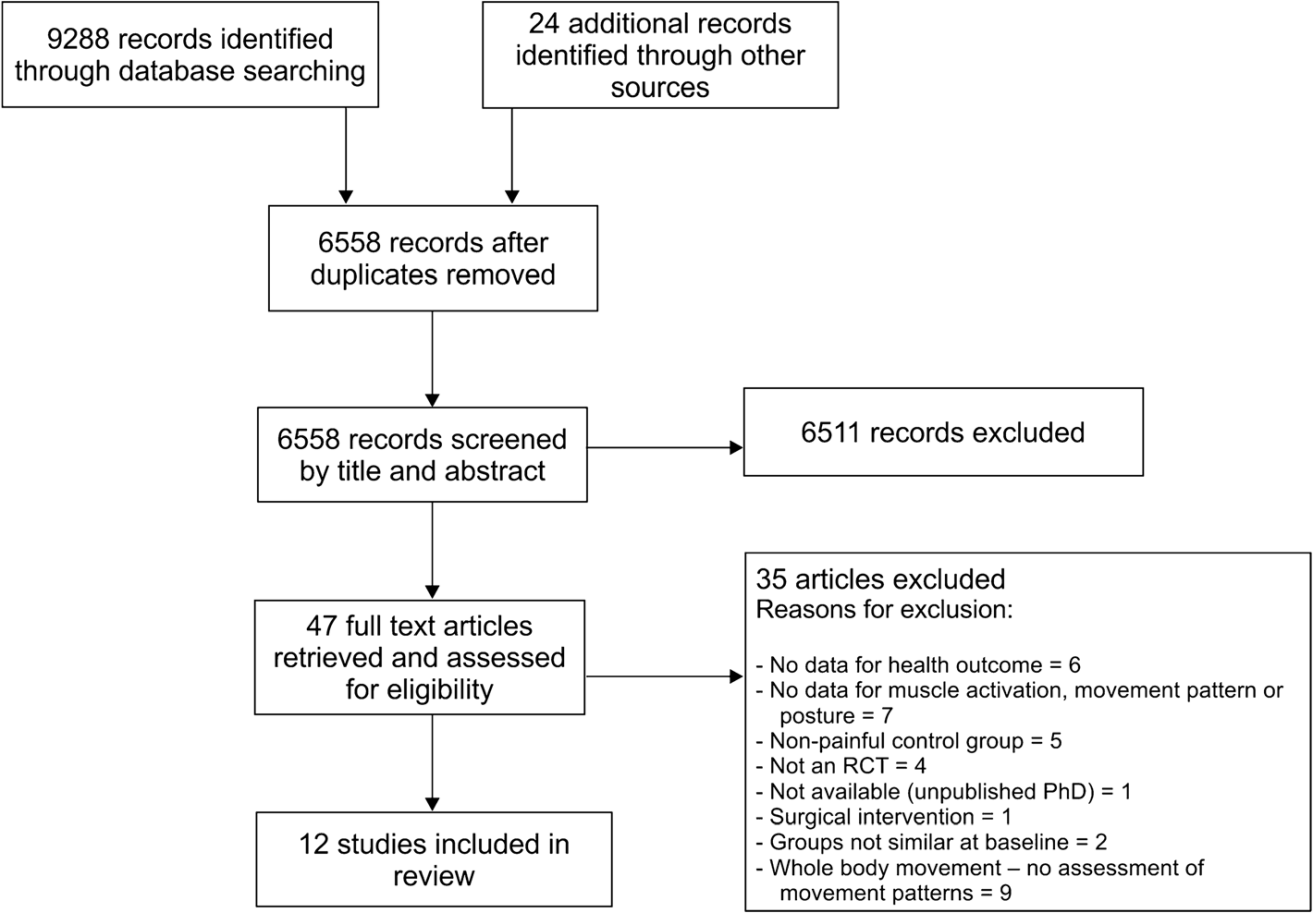
Flow diagram of study selection.

The search identified 9288 potentially relevant articles and 24 other articles were identified through other sources. Following screening of title and abstract, 47 articles were retrieved in full text. Twelve trials (16 articles) met the inclusion criteria for this review [12,18,45-47,49-60]. Most of them examined a range of physical outcome measures, however only data on patterns of muscle activity, lumbo-pelvic kinematics or posture patterns (as well as pain and activity scores) were extracted. A flow diagram of the study selection process is shown in Figure 1. The trials retrieved in full text and subsequently excluded are listed in Additional file 1, Appendix 2, together with reasons for their exclusion. Details of included studies are detailed in Additional file 1, Appendix 1. The wide variety of interventions and physical measures in the included trials prevented pooling in a meta-analysis. 

### Quality assessment

The method quality of the included trials is shown in Table 1. No trial included blinding of therapists or participants. This is not surprising, given how difficult this is to achieve in exercise or movement intervention trials. On the 0–10 quality scale, the mean score of included trials was 5.6 (range 3 to 8).

**Table 1:** Quality assessment of included studies

PEDro criteria*	**Akbari 2008**	**Da Fonesca 2009**	**Ferreira 2010**	**Haugstad 2006**	**Hides 1996&2001**	**Lalanne 2009**	**Magnussen 2008**	**Mannion 1999&2001**	**Marshall 2008**	**O’Sullivan 1997&1998**	**Ritaven 2007**	**Vasseljen 2010 , 2012 & Unsgaard-Tonsel 2010**
1. Eligibility criteria were specified	✓	✓	X	✓	✓	X	✓	✓	✓	✓	✓	✓
2. Random allocation of subjects	✓	✓	✓	✓	✓	✓	✓	✓	✓	✓	✓	✓
3. Allocation was concealed	X	X	X	X	✓	X	X	X	X	✓	✓	✓
4. Groups similar at baseline	✓	X	✓	✓	✓	✓	✓	✓	✓	✓	✓	✓
5. There was blinding of all subjects	X	X	X	X	X	X	X	X	X	X	X	X
6. Blinding of therapists	X	X	X	✓	X	X	X	X	X	X	X	X
7. Blinding of assessors	✓	X	✓	✓	✓	X	X	X	X	✓	✓	✓
8. >1 key outcome was obtained for more than 85% of subjects initially allocated to groups	X	✓	✓	X	✓	X	X	✓	✓	✓	✓	✓
9. All subjects … received the treatment or control condition as allocated or, where this was not the case, data for at least one key outcome was analysed by ‘intention to treat’	X	✓	X	X	X	X	X	X	X	X	X	✓
10. The results of between-group statistical comparisons are reported for at least one key outcome	✓	✓	✓	✓	✓	✓	✓	✓	✓	✓	✓	✓
11. The study provides both point measures and measures of variability for at least one key outcome	X	✓	✓	✓	✓	✓	X	✓	✓	✓	✓	✓
Total score	4	5	6	6	7	4	3	5	5	7	7	8
Assessor	PEDro	RL &PK	PEDro	PEDro	PEDro	PEDro	PEDro	PEDro	PEDro	PEDro	PEDro	RL &PK

* Item one is not included as part of the 10 point PEDro scoring.

### Types of trials found

Movement patterns measured by the included trials were classified into three arbitrary groups that measured: (i) specific trunk muscle activity patterns, (ii) ‘flexion relaxation response’ changes and (iii) various aspects of lumbo-pelvic kinematics and postural patterns. To focus the reporting, the analysis of results and the discussion were anchored to these three groups. Ten trials recruited people with chronic pain (> 3 months), one recruited people with both acute and chronic pain, and one recruited people with pain for less than three weeks (see Table 2 and Additional file 1).

**Table 2:** Summary of main categories of movement pattern investigated in the included studies

**Type of pattern**		**Author**	**Components of movement pattern assessed**								**Measurement details**		**Health outcomes**	
			**TA thickness**	**TA slide**	**TA + IO timing**	**LM thickness**	**Ratio muscle activitiy**	**FRR**	**Movement**	**Posture**	**Method of measurement**	**Characteristics of movement pattern measured**	**Pain**	**Activity limitation**
**Muscle activation patterns**	**Specific muscle activity**	**Akbari 2008***Motor control vs general exercise*	✓			✓					Ultrasound	Muscle size - thickness at rest (mm)	✓	
		**Hides 1996 & 2001***Motor control exercise vs medical treatment*				✓					Ultrasound	Muscle size – cross sectional area (mm^2^)	✓	
		**Ferreira 2010** motor control exercise vs general ex vs spinal manipulative therapy	✓								Ultrasound	Muscle thickness -% change from resting thickness	✓	✓
		**Marshall 2008***Swiss ball vs general exercise*			✓						Surface EMG	Feed forward activation	✓	✓
		**O’Sullivan 1997***Motor control vs GP management*					✓				Surface EMG	Internal Oblique and Rectus Abdominus electrical acitivity & ratio	✓	✓
		**Vasseljen 2010, 2012 & Unsgaard-Tonsel 2010***Motor control (low load) vs motor control (high load) vs general exercise*		✓			✓				Ultrasound	Size of muscle on contraction vs size of muscle at rest (ratio), Lateral slide (mm)	✓	✓
	**Flexion relaxation response**	**Lalanne 2009***Manipulation vs manual therapy*						✓	✓		Surface EMG and Optoelectronic recording	Angle and intensity of onset and cessation of electrical activity	✓	✓
		**Mannion 1999 & 2001***Physiotherapy vs aerobics vs devices*						✓			Surface EMG	Intensity, onset and cessation of electrical activity	✓	✓
		**Marshall 2008***Swiss ball vs general exercise*						✓			Surface EMG	Intensity, onset and cessation of electrical activity	✓	✓
		**Ritvanen 2007***Traditional bone setting vs physiotherapy*						✓			Surface EMG	Intensity, onset and cessation of electrical activity	✓	✓
**Movement patterns**		**Da Fonesca 2009***Pilates vs no Pilates control*							✓		Force plate and treadmill	Gait related forces and rates	✓	
		**Magnusson 2008***Postural biofeedback vs standardized rehab*							✓		Triaxial computerised goniometer	Circumduction area and velocity	✓	✓
**Postural patterns**		**Haugstad 2006 & 2008***Mensendieck therapy vs standard gynaelogical treatment*								✓	Visual observation	Posture, upper and lower limb movement, gait, sitting posture and respiration	✓	✓

### Trials measuring muscle activity patterns - intervention effects

Six of the 12 trials examined effects of interventions on specific muscle activity. Five trials compared motor control exercise, as described by Richardson et al. [26], with general exercise [12,47,49,56,57] and one trial compared Swiss ball exercise to general exercise [55]. Nine different outcome measures of muscle activity patterns were measured across the six trials and included TA thickness, TA movement, Lumbar Multifidus (LM) thickness, onset of contraction of the deep abdominal wall muscles and ratios of muscle activity.

Five trials (Table 3) included outcomes related to TA activity with one trial showing a statistically significant difference between experimental and comparison groups for changes to TA thickness [61] and another trial reporting a significant difference in the ratio of TA to Rectus Abdominus (RA) activity during double leg raise. [56]. No differences between groups were seen for TA movement [47] or deep abdominal wall muscle feed-forward timing [55,60]. Ferreira et al. [61] (Quality Assessment (QA) score 6/10) found significant (ANCOVA-adjusted) differences between groups in TA thickness ratio (contraction versus resting thickness) favouring motor control exercise (MCE) compared with either spinal manipulative therapy (SMT) or general exercise (GE). Effects adjusted for baseline differences were: MCE vs GE 12% greater improvement (p = 0.043); MCE vs SMT 11.4% (p = 0.053). Unadjusted post-intervention differences between groups were not significant; SMDs: MCE vs SMT −0.70 (−0.42 to 0.12); MCE vs GE −0.29 (−0.44 to 0.57). O’Sullivan et al. [57] (QA 7/10) found a significant increase in the ratio of deep (TA and Internal Oblique) to superficial abdominal wall muscle (Rectus Abdominus) EMG activity favouring the motor control group over general exercise (SMD = -0.84, 95%CI −1.47 to −0.21, p = 0.01). Hides et al. [12] (QA 7/10) reported a significant increase in Multifidus size for the motor control group compared with a medical management group but did not provide data suitable for the calculation of effect sizes. Where significant differences between groups were found, effect sizes favouring specific muscle activity (see Table 3) were small to moderate (-0.20 to −0.47), with the exception of effects observed by O’Sullivan et al. 

**Table 3:** Summary of results for studies that investigated intervention effects on muscle activity patterns (specific muscle activity)

**Muscle activity patterns (specific muscle activity)**											
**Study and intervention type (experimental vs comparison)**		**Movement pattern characteristics assessed** **Was there a statistically significant difference (p < 0.05) in physical parameters** *** between *** **groups at the end of the intervention period?*** (blank cell = not measured)*									
	**No. of Subjects**	TA thickness	TA slide*	TA & IO feedfoward timing	Multifidus (LM) thickness	Ratio of specific muscle activitiy	**Baseline differences between groups?**	SMD and 95%CIs *(negative values favour experimental/motor control group)*	Pain	Activity	SMD and 95%CIs *(negative values favour experimental group)*
**Akbari 2008***Motor control exercise vs general exercise*	49	**No**			**No**		No (TA & LM) Pain: Yes^‡^ Activity: Yes^‡^	Multifidus thickness −0.21 (−0.74 to 0.33) TA thickness −0.30 (−0.86 to 0.26)	**Yes**^‡^	**Yes**^‡^	Pain −1.06 (−1.66 to −0.46) Activity −0.70 (−1.27 to −0.12)
**Hides 1996***Motor control exercise vs control*	39				**Yes**^†**,**||^		Insufficient data	Insufficient data	**No**^†^	**No**^†^	Insufficient data
**Ferreira 2010***Motor control exercise(MCE) vs general ex (GE) vs spinal manipulative therapy (SMT)*	**34**					**Yes**^††^	**No**	TA thickness ratio (contraction vs rest) MCE vs GE −0.29 (−0.44 to 0.57)^††^ MCE vs SMT −0.70 (−0.42 to 0.12)^††^	**No**	**No**	Pain −0.32 (−0.44 to 0.54) MCE vs GE −0.51 (−0.42 to 0.30)MCE vs SMT Activity −0.25 (−1.11 to 0.61)MCE vs GE −0.63 (−0.42 to 0.19)MCE vs SMT
**Marshall 2008***Swiss ball vs general exercise*	50			**No**			No	Right feedforward activation of TA + IO −0.77 (−1.59 to 0.04 ) Left feedforward activation of TA + IO −0.46 (−1.25 to 0.34)	**No**	**Yes**	Activity −0.77 (−1.34 to −0.19)
**O’Sullivan 1997***Motor control exercise vs general exercise*	44					**Yes**	No	Ratio of TA + IO to RA −0.84 (−1.47 to −0.21)	**Yes**	No**	Pain −1.29 (−1.96 to −0.62) Activity −0.56 (−1.18 to 0.06)
**Vasseljen 2010, 2012 & Unsgaard-Tonsel 2010***Motor control (ultrasound guided exercise (US)) vs motor control (high load, sling exercise (SE)) vs general exercise (GE)*	109		**No**	**No**		**No**	No^§^	TA slide* 0.47 (−0.18 to 0.75) TA thickness ratio (contraction vs rest)^#^: TA 0.16 (−0.53 to 0.85) US vs GE IO 0.13 (−0.55 to 0.80) US vs GE EO 0.23 (−0.48 to 0.95) US vs GE TA feedforward timing:^§§^ Minimal or no effect size for most comparisons No significant feedforward differences of clinical relevance	**No**	**No**	Pain −0.46 (−1.09 to 0.18) US vs GE −0.28 (−0.90 to 0.35) US vs SE Activity −0.54 (−1.16 to 0.10) US vs GE-0.34 -0.98 to 0.30-0.01) US vs SE

TA = Transversus Abdominus, LM = Lumbar Multifidus, EO = External Oblique, IO = Internal Oblique.

* TA slide = amount of distance (mm) lateral translation of musculotendinous junction present on contraction vs relaxation.

^†^ As reported by the authors, but insufficient data for verification.

^‡^ Our calculations show a statistically significant difference between groups for pain ***and*** activity, however the groups showed a significant difference at baseline which diminishes the strength of any conclusion about relative effectiveness of the intervention.

^ §\^ No difference between groups at baseline was noted with the following exceptions: Left versus right differences were noted for the ultrasound guided group for IO ratio and TA lateral slide which created a statistically significant decrease in slide distance (reduced activation) and IO ratio post intervention for the left side only.

^||^ A statistically significant increase in favour of the experimental group for% size of Multifidus was reported by authors but insufficient data for verification.

Pain data obtained from Marshall 2008b, p331-332.

^#^ Data for US versus SE groups similar.

**Our calculations of p value differ from those reported in the study, where we calculate p = 0.076 for post intervention activity levels (difference between groups post intervention) whereas the study reports p < 0.0001. However the six-month post intervention scores do reach significance (SMD = −0.73, 95%CI −1.35 to −0.11, p = 0.021).

^††^ Authors present ANOVA data (F_2,31_ = 4.09; p = 0.026) in favour of MCE vs GE (p = 0.043) and vs SMT (p = 0.053).

^§§^ Side to side differences (nondominant versus dominant side) produced significant, small between-group differences favouring the SE group for the dominant side only (SEvs MCE and SEvs GE) after adjusting for baseline difference.

### Trials measuring muscle activity patterns - relationship between changes in muscle activity and changes in pain or activity levels

Three trials found statistically significant differences between intervention and comparison groups for pain or activity limitation. Marshall et al. [55] (QA 5/10) found no effects for measures of muscle activation but a large effect for activity limitation (but not pain) in favour of the Swiss ball group (SMD = −0.77, 95%CI −1.34 to −0.19, p = 0.06). Akbari et al. [49] (QA 4/10) compared motor control exercise to general exercise and found no significant difference between groups for TA or LM thickness but reported a positive effect for pain (SMD = −1.06, 95%CI −1.66 to -0.46, p = 0.00) and activity limitation (SMD = −0.71, 95%CI −1.28 to −0.12, p = 0.02) favouring the motor control exercise group. The treatment and comparison groups in the Akbari et al. study were significantly different at baseline (the motor control exercise group had less pain and activity limitation at baseline), confounding interpretation of intervention effects on pain and activity levels. Hides et al. [12] reported a significant difference for LM size for the motor control group when compared with the control group but no differences for pain or activity limitation. O’Sullivan et al. [56,57] reported a difference between groups favouring motor control exercise for a movement pattern characteristic (ratio of deep to superficial abdominal muscle activity) and also for pain (SMD = −1.29, 95%CI −1.96 to-0.62, p = 0.00).

### Trials measuring the flexion relaxation response - intervention effects

Four trials examined the muscle activation pattern known as the ‘flexion relaxation response’ (FRR) [51,53,55,58]. This refers to the electrical silence in lumbar extensors during full flexion typical of people without LBP; people with chronic LBP performing the same movement frequently exhibit continued electrical activity [62,63]. The FRR is a ratio where the numerator is electrical activity, measured by surface electromyography (EMG) of lumbar extensors while moving from standing to full flexion and back to standing and the denominator is EMG activity in the fully flexed position [64]. The ratio is largest in those without LBP where a normal finding would be minimal EMG activity in full flexion.

Lalanne et al. [51] (QA 4/10) compared FRR measured during a single session for people with chronic LBP who received manipulation compared with sham manipulation. They reported a significant improvement favouring the manipulation group (SMD = −1.40, 95%CI −2.24 to −0.56, p = 0.00). Marshall et al. [55] showed a significant difference in FRR favouring Swiss ball exercise over general exercise (SMD = −1.60 95%CI −2.25 to −0.94, p = 0.00). Mannion et al. [55] (QA 5/10) compared three interventions: (i) a 12-week physiotherapy group (advice, sub-maximal exercise, general strengthening, electrotherapy, heat or cold therapy, but not manual therapy), (ii) a strength training group (using devices), and (iii) an aerobics/stretching group. They found no post-intervention differences for FRR. Ritvanen et al. [60] (QA 7/10) evaluated the effects of traditional bone setting (a whole body manual therapy approach) compared with physiotherapy (massage, exercise and stretching) and found no significant post-intervention differences for FRR.

### Trials measuring the FRR - the relationship between changes to muscle activity patterns and changes to pain or activity level

No trials reporting effects on FRR found differences between groups for pain (Table 4). Marshall et al. [55] reported an improvement in FRR (SMD = −0.77, 95%CI −1.34 to −0.19, p = 0.01) and improvement in activity levels both favouring Swiss ball exercise over general exercise. 

**Table 4:** Summary of results for studies that investigated intervention effects on the Flexion relaxation response (FRR)

**Muscle activity patterns of FRR (electrical patterns of activity in extensor muscles during flexion and return from flexion) (Standardised mean difference and 95% confidence intervals, negative values favour experimental group)**								
**Study and intervention type**	**Study details**		**Movement pattern Was there a statistically significant difference (p > 0.05) in physical parameters** ***between*** **groups?**				**Health outcomes Was there a statistically significant difference (p < 0.05) in health outcomes between groups? groups?**	
	**No. of subjects**	**Baseline differences between groups?**	FRR* **Upper lumbar (T12-L3/4)**	FRR* **Lower lumbar (L4-S1)**	**Angle of onset and cessation for FRR**	**Extension vs flexion EMG ratio**	**Pain**	**Activity**
**Lalanne 2009**^‡^*Manipulation vs sham*	27	No	**Yes** ↑ -1.40 (−2.24, -0.56)	**No**	**No**	Not measured	**No**	Not measured
**Mannion 1999 & 2001***Physiotherapyvs aerobics Physiotherapy vs device strength training*	99	No	**No**^†^*Insufficient data*	**No**^†^*Insufficient data*	Not measured	Not measured	**No**	**No**
**Marshall 2008***Swiss ball vs general exercise*	50	No	**No**	**Yes** ↑ FRR in favour of intervention group −1.60 (−2.25, -0.94)	Not measured	Not measured	**No**	**Yes** Activity −0.77 (−1.34 to −0.19)
**Ritvanen 2007***Traditional bone setting vs physiotherapy*	61	(Intervention group had right vs left differences pre and post treatment)	**No**	**No** (both groups showed ↓ FRR post intervention	Not measured	**No** Trend towards increase for both groups	**No**	**No**

* *FRR = Flexion relaxation ratio (the amount of electrical activity in lumbar extensor muscles during flexion compared with end of flexion range of movement).*

^†^*As reported by authors. Insufficient data for analysis.*

^‡^*Single session intervention with pre and post analysis within session.*

### Trials measuring lumbo-pelvic kinematics and postural patterns – intervention effects

Three trials examined intervention effects on lumbo-pelvic kinematic and/or postural patterns. Measurement methods included computerised triaxial inertial goniometry [52], treadmill with a force platform [65] and visual estimation from video image recording . Haugstad et al. [51] (QA 6/10) compared Mensendieck therapy (described as a somato-cognitive movement-based therapy) with medical management for women with chronic non-specific pelvic pain. They reported significant improvement in favour of the experimental group on various physical movement and postural parameters (sitting posture and respiration post-intervention, gait and movement at 12 months) with SMDs ranging from −1.64 to −0.89 (p = 0.00 to 0.004).

Magnusson et al. [52] (QA 3/10) compared postural biofeedback with a ‘standard rehabilitation program’ in people with chronic non-specific LBP and reported a significant increase in lumbo-pelvic circumduction area but did not provide the data required to estimate effect sizes. Da Fonesca et al. [45] (QA 5/10) compared Pilates exercise with a no treatment group in a small number (n = 17) of people with chronic non-specific LBP, and found no difference between groups for gait-related parameters.

### Trials measuring lumbo-pelvic kinematics and postural patterns – relationship between changes in kinematic and postural patterns, and pain or activity levels

Haugstad et al. [50,66] reported large effects favouring Mensendieck therapy over medical management for a number of movement parameters (see Table 5) and pain (SMD = −1.71, 95%CI -2.46 to −0.97, p = 0.00). Magnusson et al. [52] reported an effect favouring postural biofeedback over a ‘standard rehabilitation program’ for movement (Table 5), pain (SMD = -3.60, 95%CI −4.5 to -2.6, p = 0.00) and activity limitation (SMD = −0.97, 95%CI -0.43 to −0.12, p = 0.00). DaFonesca et a [45] found no post-intervention difference between groups for physical parameters or pain. 

**Table 5:** Summary of results for studies that investigated intervention effects on lumbo-pelvic kinematic and postural patterns

**Lumbo-pelvic kinematic and posture patterns (Standardised mean difference and 95% confidence intervals, values favour experimental group)**									
**Study and intervention type**	**No of subjects**	**Movement pattern Was there a statistically significant difference (p > 0.05) in physical parameters** *** between *** **groups?**						**Health outcomes Was there a statistically significant difference (p > 0.05) in health outcomes between groups?**	
		**Baseline differences between groups?**	**Movement control**	**Gait**	**Standing posture**	**Respiration**	**Sitting posture**	**Pain**	**Activity**
**Da Fonesca 2009***(Pilates vs No Rx group*	17	**No**	Not measured	No*	Not measured	Not measured	Not measured	**No** −0.61, (−1.59-0.37)	Not measured
**Haugstad 2006***(Mensendieck somatocognitive therapy vs gynaecological management)*	40	**No**	**No** −0.15 (−1.29,0.98)	**No** −0.47 (−1.12,0.17),	**No** −0.20 (−0.84,0.44)	**Yes** −0.99 (−1.67, -0.31)	**Yes** −0.69 (−1.35, -0.03)	**Yes**^§^ −1.58 (−2.31,-0.85)	**Yes**^†^
**Haugstad 2008***(Mensendieck somatocognitive therapy vs gynaecological management) 12-month post intervention from Haugstad 2006*	38	**No**	**Yes** −1.07 (−1.75,-0.39)	**Yes** − 0.89 (−1.56,-0.23)	**No** −**0.56 (−1.20,0.09)**	**Yes** −1.64 (−2.38,-0.91)	**Yes** −0.99 (−1.66,-0.31)	**Yes** −1.71 (−2.46,-0.97)	**Yes**^†^
**Magnusson 2008***(Postural biofeedback vs standardised rehabilitation)*	47	**No**^||^ Insufficient data	**Yes**^‡^ Insufficient data	Not measured	Not measured	Not measured	Not measured	**Yes** −3.45 (−4.8 to −2.1)	**Yes** −0.97 (−0.43 to −0.12)

* *No difference in gait-related parameters (vertical ground reaction forces at heel strike, mid stance, toes and rate of weight acceptance) between intervention and comparison groups except for a 3% increase in mid stance for the left leg only in the Pilates group.*

^†^*Measured as part of Mensendieck score, based on averaged scores for standing posture, movement, gait, sitting posture and respiration, p < 0.000.*

^‡^*As reported by author. Insufficient data provided for analysis.*

^§^*Small baseline difference between groups p < 0.05.*

^||^*No baseline difference for pain or activity levels but insufficient data for physical parameters.*

^¶^*Calculated on the lowest number of subjects.*

## Discussion

Despite the popularity of concepts such as core stabilisation, movement normalisation and postural correction, we found only 12 trials that measured both physical change in the targeted patterns of muscle activation, lumbo-pelvic kinematics or postural patterns, and pain or activity limitation outcomes. The small number of studies available for review highlights the limited knowledge base about the ability of interventions to change movement patterns and the clinical relevance of these changes to patient-centred outcomes.

### Do interventions consistently change muscle activity patterns?

Muscle activation patterns were included in this review as they represent a specific type of movement pattern and are reportedly linked to therapeutic change with appropriate interventions Effect sizes for muscle activity pattern changes were inconsistent, mostly non-significant and generally small to moderate in size. Inconsistency may be explained by a number of factors including measurement differences. For example, Ferreira et al. [61] demonstrated significant between group differences in post intervention TA thickness favouring motor control exercise over both general exercise and spinal manipulative therapy while Vasseljen et al. [47,60], in a high quality study (QA 8/10) found no difference between motor control, sling or general exercise groups. The difference in results between these two trials may have occurred due to differences in trial method. Ferreira et al. measured right sided, unilateral TA activity following isometric knee flexion/extension while Vasseljen et al. measured bilaterally during an abdominal muscle drawing in manoeuvre. Recent evidence suggests that left and right TA can activate differentially depending on perturbation of the trunk [67]. Unilateral measurement may be insufficient to draw conclusions about TA activity and its role in movement control.

Trials that evaluated the effects of various interventions on patterns of FRR had mixed outcomes, with two trials showing significant improvements in the FRR favouring the intervention groups [51,55] and two trials showing no difference [54,58]. Methodological differences between trials may also account for these variations in results. Marshall et al. [55] demonstrated a positive change to the FRR for a group of people with chronic LBP who performed high load, Swiss ball exercise (compared with general exercise) over a three-month period, while Lalanne et al. [53] used a within-session design comparing manipulative treatment with sham treatment, that demonstrated an immediate positive change to the FRR. The very different designs and interventions confound interpretation and comparison of results. Measurement and classification differences in the calculation of the FRR further constrain comparison of these four studies. Mannion et al. [53,68] used visual assessment to grade post-intervention changes to the FRR as ‘improved, same, or worse’ while the other three trials [51,55,58] computed a ratio of electrical activity in the movement period to electrical activity in the fully flexed period but used different formulae to compute this ratio. It is possible that people with LBP may have significant variation of flexion relaxation responses. It is also plausible that not all interventions will equally affect the FRR. Dankaerts et al. [69,70] demonstrated that different patterns of muscle activation and FRR are seen in people with chronic LBP during sitting. When comparing a group of unimpaired people with people with chronic LBP, no differences were identified until people with LBP were sub-classified into groups dependent on whether flexion or extension activity provoked pain. The group classified as having pain provoked by extension showed higher lumbar extensor muscle contraction activity, while the group with pain provoked by flexion showed lower levels of muscle activity in sitting when compared with the no-pain control group. If such patterns of muscle activation, posture and movement do exist and are clinically meaningful, this could affect the results of clinical trials. In theory, a trial with a greater proportion of participants with a particular pattern of chronic LBP may have different outcomes compared to trials of participants with different patterns of muscle activation.

### The relationship of change to muscle activation patterns and changes to pain and activity limitation

The available evidence suggests little consistent relationship exists between changes to pain and/or activity level and the direction of changes to muscle activity. Changes to muscle activation patterns have been reported without corresponding change to pain or activity, while the opposite has also been reported. One could reasonably expect that if a muscle activation deficit was consistently contributing to pain or activity restriction in the broad population of people with LBP, improvements in pain and activity level would occur in conjunction with improvement in that muscle deficit. Five trials investigated changes in TA activity, with only one reporting an association between changes in TA function and associated changes in pain or activity limitation. Two trials, one involving people with acute LBP [12] and the other with chronic LBP [49], investigated Lumbar Multifidus (LM) function following motor control exercise interventions. The Hides trial [12] of people with acute LBP suggests that improvement in LM size is not directly associated with improvement in pain or activity levels. The Akbari trial [49] of people with chronic LBP that compared motor control with general exercise, found no significant post-intervention differences between groups for TA or LM size, but did find a significant improvement in pain and activity favouring the motor control group. Both the Hides and Akbari trials used ultrasound measurement of LM, which has been shown to be sensitive to changes in lumbar and abdominal muscle [71]. These findings provide preliminary evidence that changes in pain and/or activity can occur without observable change to TA or LM size and *vice versa*. O’Sullivan et al. [56,57] found a significant difference in a pattern of muscle activation (ratio of deep to superficial abdominal muscle activity), and also in both pain and activity levels, favouring motor control exercise. However the O’Sullivan et al. study differs from other studies by investigating a subgroup of chronic LBP subjects (spondylolisthesis with specific symptom pattern), while the other studies in this review included people with non-specific chronic LBP. It also differs from the other included studies with respect to the large differences observed between intervention (motor control) and control (medical management) outcomes. The improvement seen in muscle activation patterns and the related improvements in pain and activity warrant replication in another study if clinicians are to have confidence that similar outcomes would occur in the general LBP population. Recent reviews of motor control exercise for general chronic LBP populations have not concluded similar effects for pain or activity [38,39,72] and no other trials could be found that measured the ratio of deep to superficial muscle activity.

No picture emerged of a relationship between change in FRR and change in pain and activity. Marshall et al. [55] found statistically significant improvement in activity limitation favouring the experimental group. However neither of the two trials [51,55] that found improvement in FRR favouring the intervention group, were associated with any difference between groups for pain outcomes. Geisser et al. [63] in a systematic review found 11 studies comparing EMG of dynamic lumbar extensor muscle activity of people with chronic LBP with normal subjects, four of which specifically examined differences in the FRR. Based on meta-analytic pooling data from four comparable studies, they concluded that the evidence supports the FRR being a useful, measurable movement characteristic that differentiates people with LBP from people without LBP (SMD = −1.71, 95%CI −2.25 to −1.36). A recent pilot study of chronic LBP[73] showed that EMG biofeedback plus functional restoration was better than functional restoration alone in improving FRR. However the relationship between change to the FRR and changes to pain or activity limitation remains poorly explored. Increased standardisation of FRR measurement combined with a better understanding of typical variability in FRR in people with chronic LBP will be required before the implications of measuring and modifying the FRR become clear.

### Lumbo-pelvic kinematic and postural patterns

Three trials examined lumbo-pelvic kinematic and postural patterns, with only one focused on posture. The concept of changing movement or postural patterns is fundamental to many popular movement-based interventions but is rarely measured in trials of the effects of interventions. Magnusson [52] reported changes to lumbo-pelvic circumduction area favouring the postural biofeedback intervention group with associated improvements in pain and activity also favouring the intervention group. The effect sizes favouring the postural biofeedback intervention group were unusually large, and a replication study is therefore warranted. Haugstad et al. [50,66] found large and statistically significant effects in respiration and posture in favour of the intervention group using Mensendieck therapy for women with non-specific pelvic pain, as well as significant improvements in pain and activity limitation. At 12-month follow-up, the intervention group showed further improvement in movement control, gait, respiration and posture, and reduction in pain relative to the control group. In contrast, a trial by Soukup and Glomsrod [74] comparing Mensendieck therapy to a no treatment control group for people with chronic LBP found that although 12-month recurrence rates were significantly lower for the intervention group, there were no post-intervention differences between groups for pain or activity limitation. Despite a common assumption that posture is related to LBP, studies of interventions that include measurement of changes to posture are scarce, and a relationship between postural modification and improvements to pain or activity limitation has not been established.

### Measurement methods and reliability

It was beyond the scope of this review to assess the reliability of instruments used to measure movement patterns. However clinicians and researchers need to remain attentive to how movement patterns can be reliably measured and the minimal amount of change required for clinical relevance.

### Study limitations

The strengths of this systematic review are the comprehensive search strategy of a diverse selection of electronic databases, screening and data extraction by two independent reviewers. Furthermore, included studies needed to quantify a change in the targeted movement pattern so as to link that physical outcome with subsequent changes in patient-centred outcomes. The review also has limitations. Due to an absence of translation resources, only articles published in English were included and this may introduce a language, cultural and/or publication bias. The classification categories of movement patterns were necessarily arbitrary but were designed to include the most common characteristics observed in practice.

## Conclusions

This review establishes that despite the popularity of movement-related interventions, there are few clinical trials that quantify the effect of interventions for people with LBP on the outcomes of change in muscle activity, lumbo-pelvic kinematic or postural patterns. The available evidence on muscle activity pattern changes following therapeutic interventions indicates little difference in outcomes between a general exercise program and specific interventions that aim to change the activity of trunk muscles such as Transversus Abdominus and Lumbar Multifidus. That same evidence suggests that improved pain or activity limitation are consistently unrelated to changes in the activity of specific muscles. There is conflicting evidence of the effectiveness of interventions that measure changes to the flexion relaxation response, possibly due to differing trial designs and participant differences. The relationship between intervention-related change to the flexion relaxation response and changes to pain or activity limitation are also unclear. Trials of interventions that aim to change lumbo-pelvic kinematic and postural patterns are few in number, and too varied in design, to draw firm conclusions.

Overall, our ability to change movement patterns with specific interventions is not well supported by the research currently available. There is little evidence that pain and activity limitation change in concert with desirable changes to movement patterns. More research with better designs is required to advance our understanding of movement-modification through exercise.

## Abbreviations

Low back pain: LBP; Transversus abdominus: TA; Lumbar multifidus: LM; Internal oblique: IO; External oblique: EO; Standardised mean difference: SMD; Flexion relaxation response: FRR.

## Competing interests

No funding was received for this systematic review. No benefits in any form have been, or will be, received from a commercial party related directly or indirectly to the subject of this paper. This paper does not contain information about medical devices or drugs. The authors hold no stocks or shares in any company that might be directly or indirectly affected by this review. No patents have been applied for or received due to the content of this review. There are no non-financial competing interests associated with this review.

## Authors’ contributions

RL and PK contributed to data collection. RL and PK performed data inclusion and extraction with JK providing arbitration when required. All authors were involved in the design of the review, analysis and interpretation of data, drafting & revision of the manuscript, and gave approval of the final manuscript.

## Pre-publication history

The pre-publication history for this paper can be accessed here:

http://www.biomedcentral.com/1471-2474/13/169/prepub

## Supplementary Material

Additional file 1 Appendix 1. Details of included studies; Appendix 2. List of excluded studies and brief reason for exclusion. Click here for file
