# Supplementary material for: Modifying patterns of movement in people with low back pain -does it help? A systematic review
Source: BMC Musculoskelet Disord. 2012 Sep 7;13:169. doi: 10.1186/1471-2474-13-169 (PMC3466154; doi:10.1186/1471-2474-13-169)
Supplement: Additional file 1 — Appendix 1. Details of included studies; Appendix 2. List of excluded studies and brief reason for exclusion. [file 1471-2474-13-169-S1.doc]

**Addtional files:**

Appendix 1: Details of included studies

Appendix 2: List of excluded studies and brief reason for exclusion

**Appendix 1: Characteristics of included studies**

| **Study details** | **Inclusion and exclusion criteria** | **Intervention/Comparison treatments** | **Outcome measurements** | **Timing of outcome measures** |
| --- | --- | --- | --- | --- |
| **Akbari 2008**  *Source:*   - Physiotherapy clinics   *Type of condition:*   - Chronic LBP^1^   *Number of subjects:*   - N=49 post intervention (58 at baseline) | *Inclusion Criteria*   - 18-80 years old - Non specific LBP +/- leg pain, - > 3 months duration   *Exclusion Criteria*   - Serious spinal pathology - Pregnancy - Neurological compromise - Previous surgery - p. 42 ASCM guidelines^3^ | *Intervention group (n=25)*   - Muscle activation exercise program (low load activation of stabilising muscles) - 16 sessions over 8 weeks (30 min per session)   *Comparison group (n=24)*   - General exercise program - 16 sessions over 8 weeks (30 min per session) | ***Physical measures:***   - Muscle thickness (mm) of Transversus Abdominus (TA) and Lumbar Multifidus (LM) at rest   ***Measurement method***   - Ultrasound   ***Health outcomes***:   - VAS^2^ - Back performance scale (0-15 scale) | - Pre and post intervention (no details specified) |
| **Da Fonesca 2009**  *Source:*  Waiting list for physiotherapy *Type of condition:*   - Not stated   *Number of subjects:*   - N=17 (Rx group n=8, No Rx group n=9) + n=11 no pain controls, post intervention and at baseline | *Inclusion Criteria*   - Age 18-59 years old - Chronic LBP +/- leg pain > 6 months - Independent gait   *Exclusion Criteria*   - Neurological disease - Major visual deficit - True leg length discrepancy > 2 cm - Ankylosing spondylitis - Spine fusion surgery - Lower extremity surgery within 1 year | *Intervention group (N=8)*   - 15 sessions of Pilates, 2 sessions per week, (over 8 weeks but not specifically stated)   *Comparison group (n=9)*   - No treatment | ***Physical measures:***   - Parameters related to gait analysis of ground reaction forces (% body weight): - 1^st^ peak force (force of heel strike) - 2^nd^ peak force (force of toe off) - Middle support force (force mid stance) - Weight acceptance rate (left or right leg) - Push off rate   ***Measurement method***   - Force plate/treadmill   ***Health outcomes***:   - VAS - Present pain intensity (0-5 point scale) | - Pre and post intervention (no details specified) |
| **Ferreira 2010**  *Source:*  Public hospital physical therapy depts.  *Type of condition:*   - Chronic non specific LBP   *Number of subjects:*   - N=34 (Muscle activation exercise n=11, general exercise n=10, spinal manipulative therapy n=13, post intervention and at baseline) | *Inclusion Criteria*   - Age 18-80 years old - Chronic LBP +/- leg pain > 3 months - >2 on 0-10 on pain scale - >3 on Roland Morris Disability Questionnaire   *Exclusion Criteria*   - Neurological deficit - Spinal surgery previous 12 months - pregnancy - Serious or specific spine pathology - Poor English comprehension - Any contraindication to exercise | *Intervention group (N=11)*   - Average of 8.7 sessions of muscle activation exercise (Multifidus, TA, control of neutral posture + reduction on excessive superficial trunk muscle activation   *Comparison group – General exercise (n=10)*   - Average of 11.2 sessions of a program described by Klaber et al   *Comparison group – spinal manipulative therapy (n=13)*  Joint mobilisation at clinicians discretion (no high velocity thrusts) | ***Physical measures:***   - TA % thickness on contraction compared with resting thickness   ***Measurement method***   - Ultrasound   ***Health outcomes***:   - Global impression of recovery (11 point scale) - NRS - Roland Morris Questionnaire (24 item) | - Pre and post intervention (baseline and 8 weeks) |
| **Haugstad 2006**  *Source:*   - Gynaecological outpatient clinic   *Type of condition:*   - Chronic pelvic pain   *Number of subjects:*   - N=38 post intervention (40 at baseline) | *Inclusion Criteria*   - Age 20-50 - Chronic pelvic girdle pain > 1 year   *Exclusion Criteria*   - Neurological deficit or disease - Spinal canal stenosis - Lumbar disc herniation - Specific gynaecological disease - Psychological disease (bipolar, eating disorder, psychosis etc) | *Intervention group (n=19)*   - Standard gynaecological treatment (STGT) and Mensendieck somatocognitive therapy (MSCT). - Rx Group received 10 x 60 min treatments sessions with the Mensendieck therapist over 90 days (in addition to the gynaecological interventions).   *Comparison group (n=19)*   - Standard gynaecological treatment (STGT) (x2 sessions with gynaecologist for medication and advice) | ***Physical measures:***   - Mensendieck performance score (0-7 scale for each area, where 7 is best)   ***Measurement method***   - Visual observation   ***Health outcomes***:   - Visual observation (standing posture, movement, gait, sitting posture, respiration) - VAS | - Mensendieck score at baseline, 6.5 and 13 weeks - VAS and pain diary at baseline and 13 weeks |
| **Hides 1996**  *Source:*   - Emergency department, public hospital   *Type of condition:*   - Acute first episode unilateral, mechanical LBP   *Number of subjects:*   - N=39 post intervention (41 at baseline) | *Inclusion Criteria*   - Age 18-45 years - Acute 1^st^ episode unilateral LBP +/- leg referral, pain between T12 and gluteal fold, + restricted lumbar ROM   *Exclusion Criteria*   - Previous history of LBP or injury, previous lumbar surgery, spinal abnormalities indicated on radiographs, neuromuscular or joint disease, reflex and/or motor signs of nerve root compression or cauda equina compression, evidence of systemic disease, carcinoma or organ disease, pregnancy, sports or fitness training involving the low back muscles undertaken in the past 3 months. | *Intervention group (n=20)*   - Medical Rx (advice, medication) and localised, specific exercise (using ultrasound guided feedback of Multifidus) in standing, neutral position with co-contraction of TA - 4 week training   *Comparison group (n=19)*   - Medical Rx (advice, drug prescription - analgesia, NSAIDs, muscle relaxants) | ***Physical measures:***   - Muscle CSA Multifidus - Lumbar range of movement (ROM) & Straight leg raise (SLR) using double or single inclinometer   ***Measurement method***   - Ultrasound   ***Health outcomes***:   - Pain (McGill Pain Questionnaire) - VAS and pain diary - Roland Morris Questionnaire (RMQ) - ROM - Habitual activity questionnaire | - Assessed at baseline, 4 weeks and 10 weeks |
| **Lalanne 2009**  *Source:*   - Not stated   *Type of condition:*   - Chronic LBP   *Number of subjects:*   - N=27 post intervention and at baseline | *Inclusion Criteria*   - Chronic LBP > 6 months   *Exclusion Criteria*   - Spondylolisthesis, axial skeletal inflammation or osteoarthritis, collagenosis, osteoporosis, spinal surgery, neuromuscular disease, lower limb musculoskeletal injuries, malignant tumor, hypertension, infection or any other nonmechanical condition, radiculopathy, progressive neurological deficit, myelopathy, herniated lumbar disk, and severe pain (more than 7 on a 0-10 VAS scale). | *Intervention group (n=13)*   - 5 lumbar flexion-extension cycles (5 second flexion, 5 second hold, 5 second return) - Rotational manipulation   *Comparison group (n=14)*   - 5 Flexion-extension cycles 5 sec flexion, 5 sec hold, 5 sec return) - Rotational position only | ***Physical measures:***   - Electrical activity of erector spinae - Normalised RMS - Mean Flexion relaxation ratio - Angle of onset (L2, L5) - Angle of cessation (L2, L5)   ***Measurement method***   - Surface electromyography (sEMG) - Optoelectronic recording (Optotrak)   ***Health outcomes***:   - Oswestry disability index (ODI) - VAS - Fear avoidance beliefs (FABQ) | - Single intervention with pre and post analysis within session |
| **Magnusson 2008**  *Source:*   - Referral to back rehabilitation centre   *Type of condition:*   - Chronic LBP > 6 months   *Number of subjects:*   - N=47 at baseline (post intervention numbers) | *Inclusion Criteria*   - Chronic low back pain +/- leg pain - Aged 20 to 70 years - Male or female - Symptoms continuous for 6 months or more, or recurrent (not defined) - Fit for rehabilitation program   *Exclusion Criteria*   - Fracture, tumor, infection - Severe peripheral vascular disease - Symptomatic knee or hip arthritis - CNS disorders or peripheral neuropathology - Significant psychopathologic conditions | *Intervention group (n=19)*   - Standardised rehabilitation (5x1 hour sessions – advice, exercise for strengthening, posture and mobility, teaches self management strategies)   AND   - Postural biofeedback - 10 x 30 min sessions over 5 weeks   *Comparison group (n=19)*   - Standardised rehabilitation program (5 x 1 hour sessions – advice, exercise for strengthening, posture and mobility, teaches self management strategies) | ***Physical measures:***   - Lumbar ROM patterns, combined movement (circumduction) and velocity   ***Measurement method***   - Back Tracker (triaxial electrical goniometer)   ***Health outcomes***:   - VAS - SF 36 | - Baseline, 6 and 28 weeks |
| **Mannion 1999 & 2001**  *Source:*   - Community sourced via advertisement   *Type of condition:*   - Chronic LBP   *Number of subjects:*   - N=132 (See also Mannion 1999) post intervention (147 at baseline) | *Inclusion Criteria*   - < 65 years old - Chronic LBP > 3 months +/- leg pain (non radicular) - Ability to perform seated lifting 3-5kg from knee height to upright x15 in 30 sec   *Exclusion Criteria*   - Constant or persistent severe pain, nonmechanical LBP, pregnancy, previous spinal surgery, current nerve root entrap + neurologic deficit, spinal cord compression, tumors, severe structural deformity or instability or osteoporosis or cardiovascular or metabolic disease, recent fracture, inflammatory or infectious disease of the spine, other disorders preventing active rehabilitation, and lack of cooperation. | *Intervention group(s) (n=46)*   1. Physiotherapy (30 minutes, individual sessions),  - Instruction on ergonomics - Submaximal exercise (isometric and theraband) - General strength training devices - Electrotherapy (ultrasound, shortwave, TENS) - Heat/Cold treatment  1. Muscle reconditioning on training devices *(n=45)*  - 1 hour sessions in groups of 2-3, strength training, or  1. Low-impact aerobics *(n=41)*  - Twice a week for 3 months | ***Physical measures:***   - Flexion relaxation response (FRR)   *Also*   - Extensor fatigue (Biering-Sorensen) - Isometric strength trunk muscles - Lumbar ROM using CA 6000 (flexion/extension, lateral flexion, rotation)   ***Measurement method***   - Surface electromyography (sEMG)   ***Health outcomes***:   - VAS - RMQ - FABQ - Coping Strategy questionnaire | - Baseline, 13 weeks, 26 weeks |
| **Marshall 2008**  *Source:*   - Not stated   *Type of condition:*   - Chronic LBP   *Number of subjects:*   - N=50 post intervention (60 at baseline) | *Inclusion Criteria*   - CLBP non specific > 3 months duration   *Exclusion Criteria*   - Severe postural abnormality or neuromuscular disorder; previous diagnosis of pathology (confirmed by MRI or radiography), which would contraindicate exercise or spinal manipulation; manipulative treatment in the last 3 months; or previous participation in a specific abdominal stabilization training program. | 16-week intervention period, with initial 4-week self-selected Rx (manip vs non manip) followed by a 12-week randomised selection into either:  *Intervention group (n=24)*   - Specific swiss ball exercise group – weekly training, over 12 weeks   *Comparison group (n=26)*   - General home based exercise group with 3 clinic-based check-up sessions | ***Physical measures:***   - Feed-forward activation of transverse abdominal wall (Transversus Abdominus (TA) & Internal Oblique muscles (IO)) - FRR (T12/L1 and L4/5)   ***Measurement method***   - sEMG   ***Health outcomes***:   - ODI | - Baseline, 4 weeks, 8 weeks, 16 weeks, 56 weeks |
| **O’Sullivan 1997 &1998**  *Source:*   - General and specialist medical clinics, pain management and physiotherapy clinics   *Type of condition:*   - Chronic LBP in people with spondylolisthesis or spondylolysis   *Number of subjects:*   - N=42 post intervention, (44 at baseline) | *Inclusion Criteria*   - Aged between 16 – 49 years - Spondylolithesis or spondylolysis - Chronic LBP - Clinical presentation attributed to the spondylolithesis or spondylolysis by the treating medical specialist   *Exclusion Criteria*   - Previous specific stabilizsng exercise - Diagnosed psychological illness - Inadequate English comprehension - Previous spinal surgery - Diagnosed inflammatory joint disease - Presence of neurologic signs | *Intervention group (n=21)*   - Weekly sessions over 10 weeks - Training of deep abdominal muscles using abdominal drawing in manoeuvre + co-activation of lumbar Multifidi proximal to pars defect - Progression by limb loading then adding functional position   *Comparison group (n=21)*   - As directed by medical practitioner (mixed approach – general exercise, heat, massage, ultrasound, trunk curl exercises) | ***Physical measures:***   - sEMG (ratio of Internal Oblique (IO) to Rectus Abdominus (RA) activity)   *Also*   - Lx ROM (using Cybex digital inclinometer) - Hip ROM   ***Measurement method***   - sEMG   ***Health outcomes***:   - McGill pain questionnaire - ODI | - Baseline, 10 weeks,13 weeks, 26 weeks, 52 weeks |
| **Ritvanen 2007**  *Source:*   - Community sourced via advertisement   *Type of condition:*   - Chronic LBP   *Number of subjects:*   - N=61 at post intervention and at baseline | *Inclusion Criteria*   - Aged between 20 and 60 years - Chronic LBP +/- leg pain but not below knee - LBP present on at least half of the days in a 12-month period in a single episode or in multiple episodes.   *Exclusion Criteria*   - Severe neurologic, metabolic, or cardiovascular diseases, back surgery, mental diseases, a major structural abnormality (eg, kyphoscoliosis), any compensable disease, pregnancy. | *Intervention group (n=33)*   - Traditional bone setting therapy (manual whole body therapy, aiming to abolish mal positions, relax muscles, improve body symmetry) x 5 fortnightly sessions over 10 weeks   *Comparison group (n=28)*   - Physical therapy included massage, therapeutic stretching, trunk stabilisation exercise, and exercise therapy. | ***Physical measures:***   - FRR   *Also*   - Trunk ROM (finger to floor, and lateral flexion)   ***Measurement method***   - sEMG   ***Health outcomes***:   - ODI - Depression questionnaire - VAS - Patient satisfaction | - Baseline, 14 weeks |
| **Vasseljen 2010**  *Source:*   - Community sourced via advertisement and from medical practitioners   *Type of condition:*   - Chronic LBP   *Number of subjects:*   - N= 85 post intervention (109 at baseline) | *Inclusion Criteria*   - Men and women aged between 18-60years with non-specific, chronic LBP, and pain at presentation between 2 and 8 on an 11-point Numeric Rating Scale (NRS 0-10).   *Exclusion Criteria*   - Prior spinal surgery, radiating pain below the knee, other chronic pain, neurological or rheumatic diagnosis, compensable injuries, sick-leave due to LPB for more than a year at presentation, pregnancy, or insufficient comprehension of Norwegian language. | *Intervention group (n=30)*  Low load, specific exercises   - Ultrasound-guided abdominal hollowing ex (progresses to functional movements)   *Comparison groups (n=29)*   1. High load specific ex - Sling exercises 2. General exercises *(n=26)*   All groups had x1 session per week for 8 weeks | ***Physical measures:***   - Transversus Abdominus Ratio (maximum thickness on contraction divided by thickness at rest) vs Internal Oblique ratio and External Oblique ratio - Lateral slide of Transversus Abdominus (mm)   ***Measurement method***   - Ultrasound   ***Health outcomes***:   - Numerical rating scale (NRS) - Oswestry disability index - Fear avoidance belief questionnaire - BMI | - Baseline, 8-10 weeks post intervention |

^1^Chronic LBP defined as LBP> 3 months, ^2^VAS = visual analogue scale (0-10 or 0-100) for pain intensity, ^3^ American College of Sports Medicine Guidelines for exercise testing and prescription.

Appendix 2: Excluded studies and reason for exclusion

| **Study** | **Reason for exclusion (studies may have also met other exclusion criteria)** |
| --- | --- |
| Aleksiev et al, | No specific movement pattern, artificially induced movement alteration = difficult to generalise |
| Ali, | Not obtainable (PhD not published) |
| Bakhtiary et al, | No specific measure of muscle activation, no pattern assessment |
| Boston et al, | Non painful control group |
| Brox et al, | Surgical intervention |
| Childs et al, | No specific measure of muscle activation, no pattern assessment |
| Celestini et al, | No data for muscle activation, movement pattern or posture |
| Curnow et al, | No data for health outcome |
| Danneels et al, | No data for health outcome |
| Derman et al | Non painful control group |
| Dwornik et al, | No pattern of muscle activation, movement or posture measured (resting tone or gross ROM only) |
| Ferreira et al, | No specific measure of muscle activation, no pattern assessment |
| Goldby et al, | No specific measure of muscle activation, no pattern assessment |
| Harrison et al, | Unequal outcome measurement between control and treatment groups |
| Harrison et al, | Unequal outcome measurement between control and treatment groups |
| Hoffman et al, | No pain or activity data |
| Huber et al, | No pattern of muscle activation, movement or posture measured (gross ROM, EMG only) |
| Karimi et al, | Whole body movement, no health outcome measurement |
| Koumantakis et al, | Whole body movement – no assessment of patterns |
| Leinonen et al, | Whole body movement – no assessment of patterns |
| Lewis et al, | Whole body movement – no assessment of patterns |
| Lu et al, | Non painful control group |
| Luoto et al, | Non painful control group |
| Marshall et al, | Not a controlled trial |
| Mooney et al, | Non painful control group |
| Neblett et al, | Not randomised or controlled trial |
| Nouwen, | No data for health outcome |
| Petrofsky et al, | Whole body movement – no assessment of patterns |
| Poosanthanasam et al, | No data for health outcome |
| Poosanthanasam et al, | No data for health outcome |
| Roche-Leboucher et al, | No specific measure of motor control, no pattern assessment |
| Stuge et al, | No pattern of muscle activation, movement or posture measured (ASLR measured by subjective assessment) |
| Sung, | Whole body movement – no assessment of patterns |
| Suni et al, | Whole body movement – no assessment of patterns |
| Tsao et al, | No data for health outcome |

**Bibiography for excluded files**

1. Klaber Moffett J, Frost H: **Back to fitness programme. The manual for phsyiotherapists to set up the classes.** *Physiotherapy* 2000, **86**:295-305.

2. Haugstad GK, Haugstad TS, Kirste U, Leganger S, Hammel B, Klemmetsen I, Malt UF: **Reliability and validity of a standardized Mensendieck physiotherapy test (SMT)**. *Physiother Theory Prac* 2006, **22**(4):189-205.

3. Rosenstiel AK, Keefe FJ: **Coping Strategies Questionnaire (CSQ)**. *Keefe FJ, Ohio University, Athens, OH* 1983, **45701**(4).

4. Aleksiev A, Pope MH, Hooper DM, Wilder D, Magnusson M, Goel VK, Weinstein J, Spratt K, Lee S: **Pelvic unlevelness in chronic low back pain patients - Biomechanics and EMG time-frequency analyses**. *Eur J Med Rehab* 1996, **6**(1):3-16.

5. Ali AA: **Management of the first episode of acute low back pain: a comparison between two treatment protocols**. Texas Woman's University; 2002.

6. Bakhtiary AH, Safavi-Farokhi Z, Rezasoltani A: **Lumbar stabilizing exercises improve activities of daily living in patients with lumbar disc herniation**. *J Back Musculoskelet Rehabil* 2005, **18**(3-4):55-60.

7. Boston JR, Rudy TE, Lieber SJ, Stacey BR: **Measuring treatment effects on repetitive lifting for patients with chronic low back pain: Speed, style, and coordination**. *J Spinal Disord* 1995, **8**(5):342-351.

8. Brox JI, Sorensen R, Friis A, Nygaard O, Indahl A, Keller A, Ingebrigtsen T, Eriksen HR, Holm I, Kathrine Koller A *et al*: **Randomized clinical trial of lumbar instrumented fusion and cognitive intervention and exercises in patients with chronic low back pain and disc degeneration**. *Spine* 2003, **28**(17):1913-1921.

9. Childs J, Piva S, Erhard R: **Immediate improvements in side-to-side weight bearing and iliac crest symmetry after manipulation in patients with low back pain**. *J Manipulative Physiol Ther* 2004, **27**(5):306-313.

10. Celestini M, Marchese A, Serenelli A, Graziani G: **A randomized controlled trial on the efficacy of physical exercise in patients braced for instability of the lumbar spine**. *Europa Medicophysica* 2005, **41**(3):223-231.

11. Curnow D, Cobbin D, Wyndham J, Choy STB: **Altered motor control, posture and the Pilates method of exercise prescription**. *J Bodywork Movement Ther* 2009, **13**(1):104-111.

12. Danneels L, Vanderstraeten G, Cambier D, Witvrouw E, Bourgois J, Dankaerts W, De CH: **Effects of three different training modalities on the cross sectional area of the lumbar multifidus muscle in patients with chronic low back pain**. *Br J Sports Med* 2001, **35**(3):186-191.

13. Derman KL, Derman EW, Noakes TD: **A lumbar body support (KBS 2000) alters lumbar muscle recruitment patterns in patients with acute-upon-chronic lower back pain**. *S Afr Med J* 1995, **85**(4):278-282.

14. Dwornik M, Kujawa J, Bialoszewski D, Slupik A, Kiebzak W: **Electromyographic and clinical evaluation of the efficacy of neuromobilization in patients with low back pain**. *Ortopedia Traumatologia Rehabilitacja* 2009, **11**(2):164-176.

15. Ferreira ML, Ferreira PH, Latimer J, Herbert RD, Hodges PW, Jennings MD, Maher CG, Refshauge KM: **Comparison of general exercise, motor control exercise and spinal manipulative therapy for chronic low back pain: A randomized trial**. *Pain* 2007, **131**(1-2):31-37.

16. Goldby LJ, Moore AP, Doust J, Trew ME: **A randomized controlled trial investigating the efficiency of musculoskeletal physiotherapy on chronic low back disorder**. *Spine* 2006, **31**(10):1083-1093.

17. Harrison D, Cailliet R, Harrison D, Janik T, Holland B: **Changes in sagittal lumbar configuration with a new method of extension traction: nonrandomized clinical controlled trial**. *Arch Phys Med Rehabil* 2002, **83**(11):1585-1591.

18. Harrison DE, Cailliet R, Betz JW, Harrison DD, Colloca CJ, Haas JW, Janik TJ, Holland B: **A non-randomized clinical control trial of Harrison mirror image methods for correcting trunk list (lateral translations of the thoracic cage) in patients with chronic low back pain**. *Eur Spine J* 2005, **14**(2):155-162.

19. Hoffman SL, Johnson MB, Zou D, Harris-Hayes M, Van Dillen LR: **Effect of classification-specific treatment on lumbopelvic motion during hip rotation in people with low back pain**. *Man Ther* 2011, **16**(4):344-350.

20. Huber J, Lisinski P, Samborski W, Wytrazek M: **The effect of early isometric exercises on clinical and neurophysiological parameters in patients with sciatica: An interventional randomized single-blinded study**. *Isokinetics and Exercise Science* 2011, **19**(3):207-214.

21. Karimi N, Ebrahimi I, Ezzati K, Kahrizi S, Torkaman G, Arab AM: **The effects of consecutive supervised stability training on postural balance in patients with chronic low back pain**. *Pakistan Journal of Medical Sciences* 2009, **25**(2):177-181.

22. Koumantakis GA, Watson PJ, Oldham JA: **Trunk muscle stabilization training plus general exercise versus general exercise only: Randomized controlled trial of patients with recurrent low back pain**. *Phys Ther* 2005, **85**(3):209-225.

23. Leinonen V, Kankaanpaa M, Airaksinen O, Hanninen O: **Back and hip extensor activities during trunk flexion/extension: effects of low back pain and rehabilitation**. *Arch Phys Med Rehabil* 2000, **81**(1):32-37.

24. Lewis JS, Hewitt JS, Billington L, Cole S, Byng J, Karayiannis S: **A randomized clinical trial comparing two physiotherapy interventions for chronic low back pain**. *Spine* 2005, **30**(7):711-721.

25. Lu WW, Luk KD, Cheung KM, Wong YW, Leong JC: **Back muscle contraction patterns of patients with low back pain before and after rehabilitation treatment: an electromyographic evaluation**. *J Spinal Disord* 2001, **14**(4):277-282.

26. Luoto S, Taimela S, Alaranta H, Hurri H: **Psychomotor speed and postural control in chronic low-back pain patients and healthy controls - Determinants and predictive value for functional restoration outcome**. *Eur J Phys Med Rehab* 1998, **8**(3):81-86.

27. Marshall P, Murphy B: **Self-report measures best explain changes in disability compared with physical measures after exercise rehabilitation for chronic low back pain**. *Spine* 2008, **33**(3):326-338.

28. Mooney V, Gulick J, Perlman M, Levy D, Pozos R, Leggett S, Resnick D: **Relationships between myoelectric activity, strength, and MRI of lumbar extensor muscles in back pain patients and normal subjects**. *J Spinal Disord* 1997, **10**(4):348-356.

29. Neblett R, Mayer TG, Brede E, Gatchel RJ: **Correcting abnormal flexion-relaxation in chronic lumbar pain: responsiveness to a new biofeedback training protocol**. *Clin J Pain* 2010, **26**(5):403-409.

30. Nouwen A: **EMG biofeedback used to reduce standing levels of paraspinal muscle tension in chronic low back pain**. *Pain* 1983, **17**(4):353-360.

31. Petrofsky JS, Batt J, Brown J, Stacey L, Bartelink T, Le Moine M, Charbonnet M, Leyva S, Lohman EB, Aiyar S *et al*: **Improving the outcomes after back injury by a core muscle strengthening program**. *Journal of Applied Research* 2008, **8**(1):62-75.

32. Poosanthanasarn N, Lohachit C, Fungladda W, Sriboorapa S, Pulkate C, Poosanthanasarn N, Lohachit C, Fungladda W, Sriboorapa S, Pulkate C: **An ergonomics intervention program to prevent worker injuries in a metal autoparts factory**. *Southeast Asian J Trop Med Public Health* 2005, **36**(2):512-522.

33. Poosanthanasarn N, Sriboorapa S, Fungladda W, Lohachit C, Poosanthanasarn N, Sriboorapa S, Fungladda W, Lohachit C: **Reduction of low back muscular discomfort through an applied ergonomics intervention program**. *Southeast Asian J Trop Med Public Health* 2005, **36 Suppl 4**:262-270.

34. Roche-Leboucher GMD, Petit-Lemanac'h AMD, Bontoux LMD, Dubus-Bausiere VMD, Parot-Shinkel EMD, Fanello SMDP, Penneau-Fontbonne DMDP, Fouquet NS, Legrand EMDP, Roquelaure YMDP *et al*: **Multidisciplinary Intensive Functional Restoration Versus Outpatient Active Physiotherapy in Chronic Low Back Pain: A Randomized Controlled Trial**. *Spine* 2011, **36**(26):2235-2242.

35. Stuge B, Veierod MB, Laerum E, Vollestad N: **The efficacy of a treatment program focusing on specific stabilizing exercises for pelvic girdle pain after pregnancy: a randomized controlled trial**. *Spine* 2004, **29**(4):351-359.

36. Sung PS, Sung PS: **Multifidi muscles median frequency before and after spinal stabilization exercises**. *Arch Phys Med Rehabil* 2003, **84**(9):1313-1318.

37. Suni J, Rinne M, Natri A, Statistisian MP, Parkkari J, Alaranta H: **Control of the lumbar neutral zone decreases low back pain and improves self-evaluated work ability: a 12-month randomized controlled study**. *Spine* 2006, **31**(18):E611-620.

38. Tsao H, Druitt TR, Schollum TM, Hodges PW: **Motor Training of the Lumbar Paraspinal Muscles Induces Immediate Changes in Motor Coordination in Patients With Recurrent Low Back Pain**. *J Pain* 2010, **11**(11):1120-1128.
